# “Amyloid‐beta accumulation cycle” as a prevention and/or therapy target for Alzheimer's disease

**DOI:** 10.1111/acel.13109

**Published:** 2020-01-25

**Authors:** Chinthalapally V. Rao, Adam S. Asch, Daniel J. J. Carr, Hiroshi Y. Yamada

**Affiliations:** ^1^ Center for Cancer Prevention and Drug Development Department of Medicine Hematology/Oncology Section University of Oklahoma Health Sciences Center (OUHSC) Oklahoma City OK USA; ^2^ Stephenson Cancer Center Department of Medicine Hematology/Oncology Section University of Oklahoma Health Sciences Center (OUHSC) Oklahoma City OK USA; ^3^ Department of Ophthalmology University of Oklahoma Health Sciences Center (OUHSC) Oklahoma City OK USA

**Keywords:** Alzheimer's disease (AD), amyloid‐beta (Aβ), brain, cell cycle, chromosome instability (CIN), cohesinopathy, cyclin‐dependent kinase (CDK) inhibitor, mitosis, mouse, Shugoshin 1 (Sgo1)

## Abstract

The cell cycle and its regulators are validated targets for cancer drugs. Reagents that target cells in a specific cell cycle phase (e.g., antimitotics or DNA synthesis inhibitors/replication stress inducers) have demonstrated success as broad‐spectrum anticancer drugs. Cyclin‐dependent kinases (CDKs) are drivers of cell cycle transitions. A CDK inhibitor, flavopiridol/alvocidib, is an FDA‐approved drug for acute myeloid leukemia. Alzheimer's disease (AD) is another serious issue in contemporary medicine. The cause of AD remains elusive, although a critical role of latent amyloid‐beta accumulation has emerged. Existing AD drug research and development targets include amyloid, amyloid metabolism/catabolism, tau, inflammation, cholesterol, the cholinergic system, and other neurotransmitters. However, none have been validated as therapeutically effective targets. Recent reports from AD‐omics and preclinical animal models provided data supporting the long‐standing notion that cell cycle progression and/or mitosis may be a valid target for AD prevention and/or therapy. This review will summarize the recent developments in AD research: (a) Mitotic re‐entry, leading to the “amyloid‐beta accumulation cycle,” may be a prerequisite for amyloid‐beta accumulation and AD pathology development; (b) AD‐associated pathogens can cause cell cycle errors; (c) thirteen among 37 human AD genetic risk genes may be functionally involved in the cell cycle and/or mitosis; and (d) preclinical AD mouse models treated with CDK inhibitor showed improvements in cognitive/behavioral symptoms. If the “amyloid‐beta accumulation cycle is an AD drug target” concept is proven, repurposing of cancer drugs may emerge as a new, fast‐track approach for AD management in the clinic setting.

## Graphical abstract

“Amyloid‐beta accumulation cycle”. Amyloid‐beta accumulation occurs in early latent phase of Alzheimer's disease, triggering its pathology development, yet the mechanism has been elusive. This integrated hypothesis explains that amyloid‐beta accumulation occurs as a vicious cycle and offers insights on how the cycle may be interfered by cell cycle‐ or mitosis‐targeting drugs.

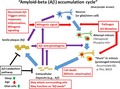

## 1 ALZHEIMER'S DISEASE REPRESENTS A MAJOR ISSUE IN CONTEMPORARY MEDICINE

Alzheimer's disease (AD) is a progressive, lethal, and incurable disease. While 3%–4% of AD is early‐onset, in which patients show cognitive symptoms in their 40s‐50s, over 96% of AD is late‐onset, in which symptoms (most notoriously, memory loss and cognitive/behavior issues) first appear at age 65 or older. In late‐onset cases, the patients live an average of four to eight years after diagnosis. As AD progresses, the patients gradually lose various social and physical functions, making disease management financially costly and emotionally burdensome for patients and caregivers. However, there has been no clinically effective medicine for AD therapy. Current FDA‐approved AD medications, such as donepezil/Aricept (central acetylcholinesterase inhibitor) and memantine (N‐methyl‐D‐aspartate [NMDA] receptor antagonist), are neuronal function modulators and can temporarily alleviate AD symptoms. These drugs do not address the pathological cause of AD and therefore do not cure AD. Unmet needs for AD drugs remain. Millions of individuals are affected by AD; for example, in the United States, 5.8 million people are living with AD in 2019 (https://www.alz.org/alzheimers-dementia/facts-figures; accessed 8/2/2019). The number of patients with AD is predicted to increase in upcoming decades, making AD an urgent issue in contemporary health care.

## 2 COMPLEX AD PATHOLOGY LEADS TO TWO MAJOR LATE‐STAGE AD BRAIN PATHOLOGIES: AMYLOID‐BETA “PLAQUES” AND TAU/P‐TAU NEUROFIBRILLARY “TANGLES”

**Figure 1:**
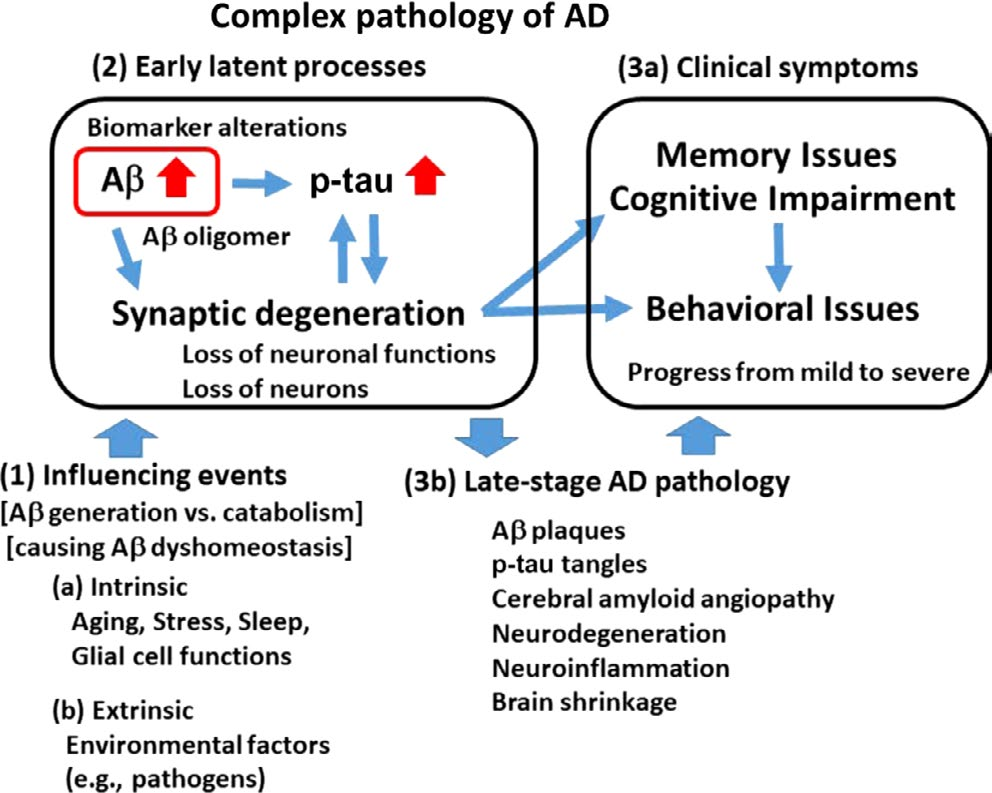
“Complex Pathology of AD”. In the clinic, AD is usually diagnosed with cognitive–behavioral symptoms and verified with brain pathology (i.e., brain scan for amyloid‐beta or observed brain shrinkage). Underlying presymptomatic latent pathologies precede the clinical symptoms by decades. Late‐stage AD patients' brains indicate amyloid‐beta plaques, p‐tau tangles, neurodegeneration, neuroinflammation, and brain shrinkage as widespread pathological traits. Understanding cellular/organ mechanisms driving the development of these pathologies (latent processes) is critical to developing drugs for AD intervention and/or therapy. Amyloid‐beta accumulation is considered a key trigger for AD pathology: (a) pathological amyloid‐beta accumulation precedes p‐tau tangles by 10–15 years (Perrin et al., 2009); (b) oligomeric amyloid‐beta is neurotoxic and can impair neuronal functions (Cline et al., 2018); and (c) amyloid‐beta catabolism is influenced by aging, stress, sleep, and glial cell functions, which is consistent with the fact that 96% of AD is late‐onset/age‐associated (see Text). Amyloid‐beta accumulation is considered a result of balance between amyloid‐beta generation (increased by stress, neuronal activity) and catabolism (which is reduced over age, helped by sleep). The mechanistic question of how amyloid‐beta begins to accumulate in middle age is a critical question

Alzheimer's disease development is accompanied by various pathological markers and events: cellular/intracellular amyloid‐beta accumulation, amyloid‐beta plaque and phospho‐tau tangle pathology, synaptic degeneration, cognitive impairment, and abnormalities in other cellular biomarkers, such as functions of the oxidative stress pathway and mitochondria. Dissecting the order of these pathology and events is critical to identifying AD drug targets (Figure 1; “Complex Pathology of AD”). Among these major pathological changes in AD brains are (a) senile “plaques” composed of an accumulation of amyloid‐beta and (b) neurofibrillary “tangles” that are mainly made of phosphorylated tau (Iqbal & Grundke‐Iqbal, 2008). Accumulated amyloid‐beta is neurotoxic and is widely considered to be the causal protein for AD development, that is, the amyloid‐beta hypothesis (Hardy & Higgins, 1992; Hardy & Selkoe, 2002; Selkoe, 2001) and its successor, the amyloid‐beta oligomer hypothesis (e.g., Cline, Bicca, Viola, & Klein, 2018). Soluble amyloid‐beta 1‐42 can form oligomers that cause synaptic failure, which leads to cognitive impairment and behavioral issues (Coleman, Federoff, & Kurlan, 2004; Klein, Stine, & Teplow, 2004). Thus, amyloid‐beta accumulation with oligomer formation is considered the triggering event for AD development, leading to cascades of events, pathway activations, and other pathologies. The amount of amyloid‐beta is a result of the balance between generation and catabolism; thus, pathways involved in amyloid‐beta generation and catabolism have been of major research interest and are candidates for AD drug targeting (Hardy & Selkoe, 2002; Selkoe & Hardy, 2016).

Amyloid‐beta is generated from the larger precursor protein amyloid precursor protein (APP) through proteolysis. Proteolytic enzymes involved in amyloid‐beta generation (i.e., the amyloidogenic pathway) include beta‐secretase (BACE) and gamma‐secretase complex (Moussa‐Pacha, Abdin, Omar, Alniss, & Al‐Tel, 2019; Penke, Bogár, & Fülöp, 2017). Amyloid‐beta can be removed through a proteolytic catabolism pathway. Sikanyika, Parkington, Smith, and Kuruppu (2019) listed 10 proteases that can catabolize Aβ1‐42. These proteases include neprilysin, endothelin converting enzyme 1, and insulin‐degrading enzyme. As the APOE4 variant increases the abundance of Aβ and risk of AD, an APOE‐associated cholesterol pathway plays a role in Aβ dyshomeostasis (Selkoe, 2001; Yamazaki, Zhao, Caulfield, Liu, & Bu, 2019). Plaques are usually surrounded by glial cells, which play a role in immunological removal of plaques (Dong, Li, Cheng, & Hou, 2019). Hence, major rational targets for AD drug R&D have been (a) amyloid‐beta itself via an immunotherapeutic approach, (b) amyloidogenic proteases, and (c) amyloid‐beta catabolism. However, these ongoing approaches have not shown success in the clinic yet. In translational studies with mouse models, a newer approach targeting amyloid‐beta oligomer has shown promise. In 6‐ to 7‐month‐old Tg 5xFAD mice, injection of an Aβ oligomer‐specific antibody rescued memory performance (Cline et al., 2018). Monomeric Aβ‐binding peptide RD2, which renders Aβ unable to form oligomer, reversed AD symptoms (cognition, behavior, and Aβ plaque loads) in aged APP/PS1 mice, indicating its potential for therapeutic application (Schemmert et al., 2019).

In late‐onset AD, amyloid‐beta accumulation and senile plaque development begin in middle age. At the early stage, no immediate visible symptoms appear, and the time window is considered presymptomatic or latent. Ten to fifteen years later, neurofibrillary tangles begin to emerge. However, there is also a 3 to 5‐year time window without notable memory or cognitive symptoms (Perrin, Fagan, & Holtzman, 2009). In a later stage of AD, memory/cognitive/behavioral symptoms and amyloid‐beta plaques and neurofibrillary tangles are observed. Notably, plaques and tangles in the AD brain appear with differential localizations; plaques are diffusely distributed over the entire cortex, while tau tangles are seen in brain regions related to clinical symptoms and overlap with areas of hypometabolism (Dronse et al., 2017). As AD progresses, both lesions spread throughout the brain, each following a stereotyped pattern. Senile plaques spread from association cortices (Thal phase 1) to allocortical areas, including the hippocampus (Thal phase 2); then to diencephalic nuclei, striatum, and cholinergic nuclei of the basal forebrain areas (Thal phase 3); several brain stem nuclei (Thal phase 4); and cerebellum (Thal phase 5) (Thal, Rüb, Orantes, & Braak, 2002). Neurofibrillary tangles first appear in the entorhinal cortex (Braak stages I/II), then spread toward limbic structures, including the hippocampus (Braak stages III/IV) and association cortices (Braak stages V/VI) (Braak & Braak, 1991). Additionally, amyloid‐beta accumulation around blood vessels, also known as cerebral amyloid angiopathy, appears.

Cerebral amyloid angiopathy may play a role in cognitive/behavioral symptoms via cerebrovascular system dysfunction (Weber, Patel, & Lutsep, 2018). This complex pathology led to a controversy regarding which pathology (amyloid‐beta plaques, tau/p‐tau tangles, or cerebral amyloid angiopathy) is more critical in AD symptoms and therefore represents a better therapeutic target. As the decades‐long development of AD pathology is associated or concurrent with many biological events and general aging, it has been difficult to pinpoint an effective intervention or therapeutic target. As approaches targeting late‐stage AD pathologies have not been successful, and as early amyloid‐beta accumulation with oligomer formation has begun to be recognized as a critical step with the amyloid‐beta oligomer hypothesis, the need to understand mechanisms leading to amyloid‐beta accumulation is increasing.

## 3 CURRENT AD DRUG RESEARCH AND DEVELOPMENT TARGETS

Mainstream AD drug research and development efforts have been directed toward (i) components directly involved in AD pathology, such as amyloid‐beta or p‐tau, amyloidogenic proteases, and other amyloid‐beta or tau binding proteins, such as receptor for advanced glycation endproducts (RAGE); (ii) components associated with pathology and predicted to be involved in symptoms, such as neuroinflammation, oxidative stress, and mitochondrial dysfunction; and (iii) medicines that can ease cognitive/behavioral symptoms of AD, including neuronal function modulators and neurotransmitters, although they may be palliative and may not address pathological causes nor lead to fundamental cure.

As of 30 October 2019, the https://Clinicaltrials.gov database lists 2,214 clinical trials involved in AD (https://clinicaltrials.gov/). The Alzforum website, which maintains a database on potential dementia therapeutics, lists 216 therapeutic reagents for AD and mild cognitive impairment (MCI) (https://www.alzforum.org/therapeutics). However, only five reagents (donepezil, galantamine, memantine, rivastigmine, and tacrine) are approved by the US FDA for use as symptomatic relievers of AD, targeting the cholinergic system and other neurotransmitters. No other agent has been validated as a clinically effective target.

## 4 INTRINSIC AND EXTRINSIC FACTORS THAT AFFECT AMYLOID‐BETA AMOUNT

The earliest step of AD pathology development is cerebral accumulation of amyloid‐beta, the mechanism of which has been elusive. A majority of sporadic AD patients do not carry mutation in familial AD genes (i.e., APP, PSEN1, or PSEN2) (Lanoiselée et al., 2017), suggesting that other drivers are involved in the development of sporadic late‐onset AD cases. Here, we discuss amyloid‐beta accumulation as a result of the disturbed balance between Aβ generation and catabolism (i.e., Aβ dyshomeostasis). Consistently, AD patients indicated impaired clearance rates for Aβ42 and Aβ40, compared with controls (Mawuenyega et al., 2010).

Various intrinsic factors can affect Aβ generation and/or catabolism. Amyloid‐beta can be generated through intensive neuronal activity (i.e., activity‐dependent Aβ generation) (Ovsepian & O'Leary, 2016). Activation of and/or increase in amyloidogenic proteases can play a role in increasing Aβ, as observed in patients with Down syndrome who exhibit early AD‐like dementia and express a large amount of BACE1 (Miners, Morris, Love, & Kehoe, 2011). Systemic factors, such as the effect of oral and gut microbes on systemic inflammation, are also gathering interest (Tremlett, Bauer, Appel‐Cresswell, Finlay, & Waubant, 2017). Sleep affects accumulation and removal of Aβ (Cordone, Annarumma, Rossini, & Gennaro, 2019). Reports indicate that Aβ catabolism decreases over age. Older mice showed a 40% decline in clearance of injected Aβ, which may be caused by a decline in the efficiency of exchange between the subarachnoid cerebrospinal fluid and the brain parenchyma (Kress et al., 2014). Levels of neprilysin, an Aβ‐degrading protease, decreased with age in both normal and AD patients. This decreasing neprilysin level may act as a trigger for AD (Hellström‐Lindahl, Ravid, & Nordberg, 2008). In APP‐SL70 mice, the microglial response to increasing amyloid‐beta was estimated to be overwhelmed with aging (Blume et al., 2018).

In addition to intrinsic factors, extrinsic or environmental factors can play a role. In a mouse model, recurrent activation of brain herpes simplex virus 1 (HSV1) infection led to amyloid‐beta accumulation and other AD pathology (tau phosphorylation, neuroinflammation) (De Chiara et al., 2019), indicating that viral infection can trigger Aβ accumulation and AD pathology. In an AD‐omics study, HHV‐6A and HHV‐7 were identified as prominently associated with human AD across three independent cohorts (Readhead et al., 2018). Reports like these support the theory that pathogens trigger AD (Haas & Lathe, 2018; Itzhaki, 2014; Sochocka, Zwolińska, & Leszek, 2017), as well as the role of amyloid‐beta as a protection mechanism against viral infection (Li, Liu, Zheng, & Huang, 2018). Although AD cannot be completely explained by the pathogen theory alone, pathogens may act as a risk factor or have an impact on a segment of patients with AD. A high rate of HSV1 and other infections was observed in AD patients (Sochocka et al., 2017). Since three subtypes of AD were identified based on the spread of neurofibrillary tangles (Murray et al., 2011), there may be more than one causal process, leading to distinct pathology of AD subtypes.

## 5 A BRIEF HISTORY OF EARLIER STUDIES OF ANEUPLOIDY AND AD

Since the 1990s, a long‐standing theory has purported that aneuploidy plays a critical role in AD development. In an early thesis noting AD‐like dementia in patients with Down syndrome, Potter (1991) hypothesized that (i) aneuploidy (chromosome 21 trisomy) is causal to AD, and that (ii) genes associated with the risk of AD would be involved in the cell cycle, and such genes would lead to the development of aneuploidy when mutated (Potter, 1991). Following hypothesis (i), the link between aneuploidy and AD was explored using cytogenetics. Earlier studies tested the rate of aneuploidy in peripheral blood lymphocytes and fibroblasts from AD patients. Cells from familial and sporadic AD patients were shown to have more micronuclei than controls. The antifungal drug griseofulvin mitigated the increase in micronuclei in cells from patients with AD, indicating an altered response to genotoxic challenge (Petrozzi et al., 2002; Trippi et al., 2002). These results may be attributed to increased DNA damage and impaired DNA repair (Coppedè & Migliore, 2009), or perhaps to DNA replication stress (Yurov, Vorsanova, & Iourov, 2011). Newer studies indicated a higher aneuploidy rate in AD‐affected neurons (Iourov, Vorsanova, Liehr, & Yurov, 2009). In addition, the presence of hyperploid neurons was noted (Arendt, Brückner, Mosch, & Lösche, 2010) [see Section 6.3 “High degree of aneuploidy in patients with AD and mild cognitive impairment (MCI)” for additional references]. Although a single‐cell sequencing report noted conflicting results (van den Bos et al., 2016), and although reported rates of aneuploidy vary widely, collectively there seems to be solid support for increased aneuploidy in somatic cells and neurons of patients with AD. Supporting evidence for hypothesis (ii) includes reports that gene mutations causal to neurodegenerative diseases (APP, MAPT, PSEN for AD [see later sections]; Niemann–Pick C; Granic & Potter, 2013) also cause aneuploidy in neurons. In a later section, “Thirteen among 37 genes on the human AD genetic risk loci are functionally involved in the cell cycle and/or mitosis” (Table 1), we will discuss newer corroborating evidence from contemporary AD‐omics.

**Table 1:** Thirteen among 37 genes on human AD genetic risk loci are functionally involved in the cell cycle and/or mitosis

Gene Name	Full Name	Proposed function (s) and pathway(s) involved*	“Cell cycle”/total publications**	“Mitosis”/total publications**	Reported involvement in cell cycle and/or mitosis (including possible link)
ABCA7	ATP‐binding cassette subfamily A member 7	Transporter, Lipid metabolism/ homeostasis, Ceramide transport	2/228	1/228	Ovarian cancer metastasis marker candidate (Elsnerova et al., 2017)
**ABI3**	ABI family member 3	Inhibits ectopic metastasis of tumor cells and cell migration	13/406	1/406	Tumor suppressor, Expression reduces growth and induces senescence (Latini et al., 2011)
ACE	Angiotensin I‐converting enzyme	Generates angiotensin II, a potent vasopressor and aldosterone‐stimulating peptide that controls blood pressure and fluid–electrolyte balance	126/13779	6/13779	Inhibition accelerates endothelial regrowth (Van Belle et al., 1997)
ADAM10	ADAM metallopeptidase domain 10	Metalloproteinase, Cleaves the membrane‐bound precursor of TNF‐alpha to its mature soluble form, Cleaves several other cell‐surface proteins (e.g., ephrin‐A2, CD44, CDH2, Notch)	32/1364	1/1364	Silencing inhibits the in vitro and in vivo growth of hepatocellular carcinoma cells (Liu, Zhang, Liu, Ji, & Wang, 2015), Constitutive activation promotes cell growth and activates the TNF‐α/NFκB pathway in mantle cell lymphoma (Armanious, Gelebart, Anand, Belch, & Lai, 2011)
ADAMTS4	ADAM metallopeptidase with thrombospondin type 1 motif 4	ADAMTS (a disintegrin and metalloproteinase with thrombospondin motifs) protein family, Cleaves aggrecan, a cartilage proteoglycan, and may be involved in its turnover	6/578	0/578	
ALPK2	Alpha kinase 2	Kinase that recognizes phosphorylation sites in which the surrounding peptides have an alpha‐helical conformation	0/13	0/13	
APH1B	Aph−1 homolog B, gamma‐secretase subunit	Functional component of the gamma‐secretase complex, which also contains presenilin and nicastrin, a subunit of APP protease complex	1/37	0/37	
**BIN1**	Bridging integrator 1	Nucleocytoplasmic adaptor, synaptic vesicle endocytosis, cardiac muscle development	35/413	0/413	Tumor suppressor (Pan et al., 2012), A corepressor of the transcription factor E2F1, Inhibits cell cycle progression (Folk et al., 2019), Regulates fas/fas ligand‐mediated apoptosis (Esmailzadeh, Huang, Su, Zhou, & Jiang, 2015), Accumulates adjacent to amyloid deposits in vivo (De Rossi et al., 2019)
BZRAP1‐AS1	BZRAP1 antisense RNA 1	Noncoding RNA, Promoters and enhancers for TSPOAP1‐AS1 gene	0/3	0/3	
CASS4	Cas scaffold protein family member 4	Docking protein that plays a role in tyrosine kinase‐based signaling, related to cell adhesion and cell spreading	3/25	0/25	
CD33	CD33 molecule	Lectin of the SIGLEC (Sialic acid‐binding immunoglobulin‐like) family, cell–cell interaction, Transmembrane receptor expressed on cells of myeloid lineage	241/3168	4/3168	Marker for acute myeloid leukemia subtype, CD33 inhibition in myeloid cells causes apoptosis (Mingari, Vitale, Romagnani, Falco, & Moretta, 2001), Anti‐CD33 antibody conjugates are being tested for CD33 + AML in clinic (Kobayashi et al., 2009).
**CD2AP**	CD2‐associated protein	Scaffolding molecule that regulates the actin cytoskeleton, receptor endocytosis, and cytokinesis	17/403	1/403	Involved in cytokinesis (Monzo et al., 2005)
**CELF1**	CUGBP Elav‐like family member 1	May regulate pre‐mRNA alternative splicing, mRNA editing, and translation; May be a specific regulator of miRNA biogenesis; Defects affect myotonic dystrophy via RNA toxicity	21/234	0/234	Upregulated in glioma, Promotes glioma cell proliferation by suppression of CDKN1B (Xia et al., 2015)
CLNK	Cytokine‐dependent hematopoietic cell linker	Regulation of immunoreceptor signaling	0/22	0/22	
**CLU**	Clusterin (aka. apolipoprotein J)	A chaperon (secreted and cytosolic), Inhibits formation of amyloid fibrils, Involved in cell death, tumor progression, and neurodegenerative disorders	119/1759	7/1759	CLU OP activates PI3K/AKT pathway, overrides Cr(VI)‐induced senescence in hepatocytes (Zhang, Zhang, Xiao, Zhong, and Xiao, 2019), CLU knockdown sensitizes cancer cells to chemotherapy drugs (Al Nakouzi et al., 2016), Nuclear CLU is pro‐apoptotic (Shannan, Seifert, Boothman, Tilgen, & Reichrath, 2006), Secretory CLU is pro‐survival, High levels of sCLU caused G1 cell cycle arrest in distinct cell types (Yu & Tan, 2012)
CNTNAP2	Contactin‐associated protein‐like 2	Cell adhesion molecules and receptors in nervous system	3/395	0/395	
CR1	Complement C3b/C4b receptor 1	Membrane immune adherence receptor, Belongs to the receptors involved in complement activation, Captures and clears complement‐opsonized pathogens by erythrocytes and monocytes/macrophage	88/3263	5/3263	
**DSG2**	Desmoglein 2	Calcium‐binding transmembrane glycoprotein components of desmosomes and cell–cell junctions	13/310	0/310	Overproduction is poor prognostic marker for HCC (Han et al., 2018), Knockdown arrests NSCLC cells via CDK2 decrease and p27 increase (Cai et al., 2017)
ECHDC3	Enoyl‐CoA hydratase domain‐containing 3	Fatty acid biosynthesis	0/12	0/12	
**EPHA1**	EPH receptor A1	Ephrin receptor subfamily of the protein tyrosine kinase, Nervous system development, Contact‐dependent bidirectional signaling into neighboring cells	16/246	1/246	Negative regulator of the Ras/MAPK pathway (Miao et al., 2001), exerts antimitogenic functions in a cell‐type‐specific manner, Knockdown of EPHA1 in ovarian cancer cells inhibited their aggressive traits
FERMT2	Fermitin family member 2	Scaffolding protein, Enhances integrin‐mediated cell adhesion onto the extracellular matrix and cell spreading, Binds to membranes enriched in phosphoinositides, the assembly of focal adhesions	4/64	0/64	Inhibited by Wnt/beta‐catenin, resulting in blockade of myoblast fusion in myoblasts (Suzuki, Pelikan, & Iwata, 2015)
HESX1	HESX homeobox 1	Conserved homeobox protein that is a transcriptional repressor in the developing forebrain and pituitary gland	3/235	0/235	
HLA‐DRB5, HLA‐DRB1	Major histocompatibility complex, class II, DR beta 5, and DR beta 1	Plays a central role in the immune system by presenting peptides derived from extracellular proteins	0/464 (DRB5) 60/9178 (DRB1)	0/464 (DRB5) 0/9178 (DRB1)	
**INPP5D**	Inositol polyphosphate−5‐phosphatase D	Hydrolyzes the 5' phosphate from phosphatidylinositol (3,4,5)‐trisphosphate and inositol 1,3,4,5‐tetrakisphosphate, negatively regulating the PI3K (phosphoinositide 3‐kinase) pathways, Negative regulator of myeloid cell proliferation and survival	7/288	0/288	Overexpression suppressed cell growth, migration, and invasion in vitro and in vivo in NSCLC via PI3K pathway inhibition (Fu et al., 2019)
**KAT8**	Lysine acetyltransferase 8	Histone acetylase (HAT), Chromatin architecture, Embryonic development	32/145	3/145	Important for cancer cell survival (Zhang, Liu, et al., 2013), Required for DNA damage response and double‐strand break repair to ionizing radiation, RNAi for Rcd1, Rcd5, or MBD‐R2 showed abnormal chromosome segregation in Drosophila (Pavlova et al., 2019)
**MEF2C**	Myocyte enhancer factor 2C	MADS box transcription enhancer factor 2 (MEF2) family, Controls cardiac morphogenesis and myogenesis, Essential role in hippocampal‐dependent learning and memory by suppressing the number of excitatory synapses and thus regulating basal and evoked synaptic transmission, Normal neuronal development	65/1122	4/1122	Regulates the expression of G2/M checkpoint genes (14‐3‐3γ, Gadd45b, and p21) and the subcellular localization of CYCLIN B1 (Badodi, Baruffaldi, Ganassi, Battini, & Molinari, 2015), Substrate of anaphase‐promoting complex, Expression of phosphorylation mutant can delay cell cycle in colon cancer cells, Activates CDK inhibitor p21/CDKN1A and thus inhibits cell cycle transition (Di Giorgio, Gagliostro, Clocchiatti, & Brancolini, 2015), Acts as effectors of neurogenesis in the brain (Li et al., 2008), Drives B‐cell receptor (BCR)‐induced proliferation of mature B cells (Wilker et al., 2008)
MS4A6A MS4A4E	Membrane spanning 4‐domains A6A, A4E	May be involved in signal transduction as a component of a multimeric receptor complex	1/60 (MS4A6A) 1/26 (MS4A4E)	0/60 (MS4A6A) 0/26 (MS4A4E)	
NME8	NME/NM23 family member 8	Ciliary function, Sperm tail maturation	0/31	0/31	
NYAP1	Neuronal tyrosine‐phosphorylated phosphoinositide−3‐kinase adaptor 1	Regulates neuronal morphogenesis, Disruption in mice affects brain size and neurite elongation	0/4	0/4	
PTK2B/PYK2	Protein tyrosine kinase 2 beta	Calcium‐induced regulation of ion channels and activation of the map kinase signaling pathway, Member of the FAK subfamily of protein tyrosine kinases	23/457	4/457	Regulates actin cytoskeleton reorganization in fibroblasts (Du et al., 2001), p‐PYK2 associates with the oocyte spindle and spindle poles, May function as a component of the microtubule‐organizing center to regulate spindle assembly during the meiotic process of mouse oocytes (Meng et al., 2018), Promotes migration and invasion of glioma cells (Lipinski et al., 2005)
SCIMP	SLP adaptor and CSK interacting membrane protein	Transmembrane adapter/mediator, Major histocompatibility complex class II signal transduction, Immune synapse formation	0/9	0/9	
SLC24A4	Solute carrier family 24 member 4	Potassium‐dependent sodium/calcium exchanger protein family, transporter	0/84	0/84	
SORL1	Sortilin‐related receptor 1	Endocytosis and sorting, Transmembrane signaling receptor activity and low‐density lipoprotein particle binding	4/334	2/334	
**SPI1/PU.1**	Spi−1 proto‐oncogene, hematopoietic transcription factor PU.1	ETS‐domain transcription factor that activates gene expression during myeloid and B‐lymphoid cell development, Binds to the PU‐box, a lymphoid‐specific enhancer, Differentiation or activation of macrophages or B cells	145/1961	4/1961	PU.1 is essential for mixed‐lineage leukemia (MLL) (Zhou et al., 2014), Required for the growth of MLL leukemic cells via the promotion of cell cycle progression and inhibition of apoptosis, Acts as tumor suppressor in myeloma (Ueno et al., 2017)
**SUZ12P1**	SUZ12 pseudogene 1		0/1	0/1	Prostate cancer biomarker, Long noncoding RNA promoted the proliferation of and inhibited apoptosis of prostate cancer (Wan et al., 2016)
**TREM2**	Triggering receptor expressed on myeloid cells 2	Immune response, Membrane protein that forms a receptor signaling complex with the TYRO protein	9/708	0/708	Promotes microglial survival by activating the Wnt/β‐catenin pathway (Zheng et al., 2017), TREM2/DAP12 complex regulates inflammatory responses in microglia via the JNK signaling pathway (Zhong et al., 2017), Overexpression enhances glioma cell proliferation and invasion (Wang et al., 2016). Acts as a tumor suppressor via Wnt1/β‐catenin and Erk signaling in colorectal carcinoma (Kim et al., (2019)
ZCWPW1	Zinc finger CW‐type and PWWP domain‐containing 1	Function poorly known, A “bottleneck” gene	0/32	0/32	

Note

A list of 37 AD genetic risk loci (genes identified as frequently mutated in AD‐omics studies) was used. The number of existing publications was examined for each gene, followed by a keyword search with “Gene X and cell cycle” or “Gene X and mitosis.” The process provides an estimate for the current total research activity regarding the gene and for the gene's functional involvement in the cell cycle and/or in mitosis. Genes showing a direct or strong functional connection to the cell cycle and/or mitosis are marked in bold. Thirteen among the 37 AD genetic risk loci have indicated functions in the cell cycle and/or mitosis, suggesting the importance of the cell cycle and/or mitosis in AD development.

* Based on GeneCards database.

** Publication numbers as of 5 November 2019, via PubMed keyword search.

In Boveri's 19th‐century theorem, aneuploidy was predicted to cause cancer (Boveri, 2008). Aneuploidy can be caused by external genotoxic challenges (e.g., radiation, chemicals, and virus) or by an internal defect in molecular mechanisms for genome maintenance that are intimately involved in cell cycle regulations. Mainstream mechanistic studies of the cell cycle emerged rather independently from studies of AD. The conceptual framework that the cell cycle is driven by cyclin‐dependent kinases (CDKs) and cyclins emerged by the mid‐1980s/early 1990s (Nurse, 2012). During the same period, major cell cycle driving enzymes and their regulatory components were identified through combined efforts, including genetics work using budding yeast *S. cerevisiae*, fission yeast *S. pombe*, fungi *A. nidulans*, and fruit fly *D. melanogaster*; biochemistry studies using egg extracts of frog *X. laevis*; and cell biology studies with cultured cells (see Murray & Hunt, 1993).

Cancer, carcinogenesis, and developmental defects associated with aneuploidy have been the primary disease targets of cell cycle studies. These types of investigations were assumed to be useful for finding methods and targets to manipulate cell cycle and cell growth, which could lead researchers to cancer therapeutics. Over the years, this assumption has been shown to be correct. Many validated cancer therapeutics target machineries that are involved in the cell cycle. In the 1990s–2000s, aneuploidy‐inducing transgenic genomic instability mouse models, such as chromosome instability (CIN) models and microsatellite instability (MIN) models, were developed. The models were used mainly to assess cancer development (Rao & Yamada, 2013; Rao, Yamada, Yao, & Dai, 2009; Simon, Bakker, & Foijer, 2015) and to examine the relationship between aneuploidy and carcinogenesis (Weaver & Cleveland, 2009; Zasadil et al., 2016). However, most cancer assessment studies do not keep mice until old age. It was only recently that experimental results testing the link between genomic instability and AD in aged mouse models started to be reported (Rao, Farooqui, Asch, & Yamada, 2018).

## 6 THE “AMYLOID‐BETA ACCUMULATION CYCLE”

**Figure 2:**
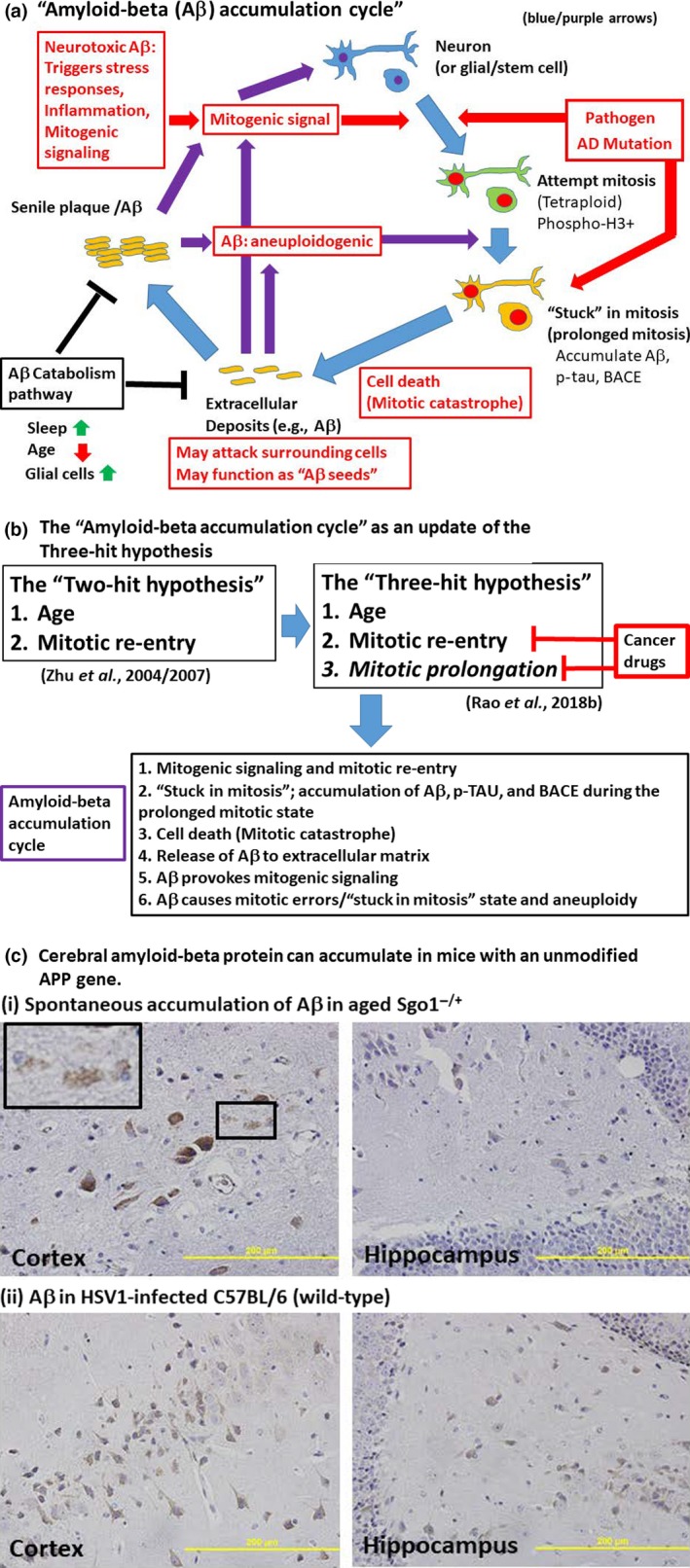
(a) The “amyloid‐beta accumulation cycle”. Normal neurons or glia are challenged by mitotic signaling, which may be associated with age and the microenvironment, such as high reactive oxygen species (ROS), reduced antioxidants, damaged blood–brain barrier, and fatigued stem cells, or other pathogenic conditions, such as diabetic wounds, pathogen infection, or mutated AD risk gene. Mitogenic signaling causes neurons or glial cells to enter the cell cycle and attempt to go through mitosis. In the cycling cells, aneuploidy, an environmental factor, other mutations in an AD risk gene, or already existing extracellular amyloid‐beta cause cells to go through prolonged mitosis or a quasi‐mitotic state with high mitotic kinase activity, when they accumulate amyloid‐beta, BACE, and p‐tau. If the state is not resolved, mitotic catastrophe occurs, and accumulated amyloid‐beta, BACE, and p‐tau are released to the microenvironment. Released amyloid‐beta, with its prion‐like properties, may function as seeds for subsequent plaque pathology. Extracellular amyloid‐beta can provoke inflammation and mitogenic signaling, and can also cause mitotic errors, prolonged mitosis, and aneuploidy. Thus, age‐ or microenvironment‐provoked mitogenic signaling can trigger a vicious cycle leading to further amyloid‐beta accumulation (the “amyloid‐beta accumulation cycle”) (blue/purple arrows). (b) Cancer drugs that target mitotic re‐entry and/or prolonged mitosis may be valid drugs for managing the “amyloid‐beta accumulation cycle” and AD. The two‐hit hypothesis (Zhu et al., 2007, 2004) proposed age and mitotic re‐entry as crucial events for development of AD pathology. In light of the apparent importance of prolonged mitosis in this process, we proposed the three‐hit hypothesis (Rao, Farooqui, Asch et al., 2018). The “amyloid‐beta accumulation cycle” is an integrated hypothesis that emerged from the three‐hit hypothesis. The “amyloid‐beta accumulation cycle” suggests that a reagent that interferes with amyloid‐beta accumulation could be an AD drug. As the cell cycle and mitosis are validated targets for cancer drugs, repurposing of cancer drugs for AD management may emerge as a viable clinical option in the near future. (c) Cerebral amyloid‐beta protein can accumulate in mice with an unmodified APP gene under certain conditions. Under normal circumstances, wild‐type mice with an unmodified APP gene do not accumulate amyloid‐beta in the brain, even in old age (24 months and older). AD modeling in mice has been dependent on introduction of transgenic mutations in genes involved in familial/early‐onset AD (e.g., APP, PSEN1, and MAPT), representing early‐onset AD models (Jankowsky & Zheng, 2017; Saito & Saido, 2018). A rodent model for sporadic late‐onset AD has been an unmet need. Over 96% of all human AD cases are late‐onset and sporadic, a majority of which carry no mutation in known early‐onset AD genes. Thus, identifying conditions under which amyloid‐beta accumulates is valuable to gain mechanistic insights on AD development and to model late‐onset AD. A progeria mouse model SAMP8 was reported to accumulate amyloid‐beta, yet the causal mutation remains unidentified (Akiguchi et al., 2017). Recent reports began to identify conditions that can cause amyloid‐beta accumulation in the mouse brain with unmodified APP or other known early‐onset AD gene mutations. Examples of amyloid‐beta accumulating conditions include (i) aged Sgo1^−/+^ mice, a cohesinopathy–chromosome instability mouse model (Rao, Farooqui, Zhang et al., 2018) (photo: Our Aβ IHC results from 18‐ to 24‐month‐old Sgo1^−/+^ mice. The magnified panel indicates extracellular “released” Aβ), and (ii) HSV1 infection (e.g., De Chiara et al., 2019). Photo: Our Aβ IHC results from HSV1‐infected 12‐month‐old C57BL/6 mice (unpublished). Uninfected mice showed no cerebral amyloid‐beta (not shown). Antibody used for IHC: Cell Signaling Technology β‐Amyloid D54D2 (cat. No. 8243). Although multiple Aβ‐specific commercial antibodies recognized the same band, the exact Aβ species accumulated in Sgo1^−/+^ model remain to be determined

From the notion that aneuploidy plays a role in AD development, some hypotheses focusing on the role of mitotic cycle re‐entry of neurons evolved. One such hypothesis was the “two‐hit hypothesis” that proposes age and mitotic re‐entry as two key factors (“hits”) for AD development (Webber et al., 2005; Zhu, Lee, Perry, & Smith, 2007; Zhu, Raina, Perry, & Smith, 2004). Based on results from genomic instability mouse models, we proposed a version of the two‐hit hypothesis with an emphasis on the role of prolonged mitosis in accumulating amyloid‐beta, the “three‐hit hypothesis.” The “three‐hit hypothesis” proposes (I) aging, (II) mitotic re‐entry, and (III) prolonged mitosis as three key factors for the development of AD (Rao, Farooqui, Zhang, Asch, & Yamada, 2018). Recent literature led us to an integrative hypothesis, the “amyloid‐beta accumulation cycle,” incorporating interference in mitosis and the aneuploidogenic role of amyloid‐beta (Figure 2). The rationales for the “amyloid‐beta accumulation cycle” are summarized below.

### 6.1 Neuronal cell cycle re‐entry occurs in AD

Normally, neurons are terminally differentiated cells and maintain their G0 quiescent state. However, human AD brains show signs of neurons in the mitotic cycle and poly(tetra)ploid 4N cells (Herrup, 2010; Herrup & Arendt, 2002; Herrup, Neve, Ackerman, & Copani, 2004; Neve & McPhie, 2006). Consistently, human AD brains show misregulations in canonical cell cycle driving proteins (e.g., CDC25 phosphatases) and inhibitory proteins (e.g., wee1 kinase) in the direction toward mitosis; in degenerating neurons, Cdc25A and Cdc25B show higher activity, while Wee1 shows lower activity (Ding et al., 2000; Tomashevski, Husseman, Jin, Nochlin, & Vincent, 2001; Vincent et al., 2001). In a 3xTg AD mouse model and in human AD patients, hyperphosphorylated retinoblastoma protein, a marker for G1/S transition, co‐localized with hyperphosphorylated tau, linking aberrant cell cycle progression with tau pathology (Hradek et al., 2015). In the study, Hradek et al. used 19‐month‐old animals, thus leaving a question if the pRb accumulation is age‐associated. Lopes, Blurton‐Jones, Yamasaki, Agostinho, and LaFerla (2009) used two mouse models, (i) 3xTg and (ii) a tetracycline‐regulatable transgenic model of neuronal ablation (CaM/Tet‐DTA mice). Neuronal death in model (ii) did show increases in cell cycle markers, suggesting that cell death and release of cellular contents can activate mitogenic signaling. This model also displayed a significant increase in hyperphosphorylated tau and Abeta, supporting the possibility that cell cycle re‐entry may lead to AD‐like changes even in animals without a previous alteration of the genes related to Abeta or tau.

### 6.2 Various mitogenic/growth signaling factors are activated in the AD brain

Consistent with Section 6.1, misregulations in various mitogenic/growth signaling factors, including ERK/MAPK, GSK3, PI3K/AKT, and CDK, are reported in the AD brain (Kirouac, Rajic, Cribbs, & Padmanabhan, 2017; Monaco & Vallano, 2005; Morroni, Sita, Tarozzi, Rimondini, & Hrelia, 2016; Sun, Liu, Nguyen, & Bing, 2003; Swatton et al., 2004; Vincent, Jicha, Rosado, & Dickson, 1997). Amyloid‐beta oligomers are synaptotoxic and can activate the signaling axis that involves the tyrosine kinase ephrin receptor A4 (EphA4) and c‐Abl tyrosine kinase. When mutated, c‐Abl can act as an oncogene (Vargas, Cerpa, Muñoz, Zanlungo, & Alvarez, 2018). CDK7 can act as a CDK‐activating kinase and can activate the major Cdk–cyclin substrates. CDK7 expression is age‐dependent and is elevated in hippocampal neurons of AD patients (Zhu et al., 2000). The stress signaling AMPK pathway is also reported to be involved in AD (Caberlotto, Lauria, Nguyen, & Scotti, 2013; Mairet‐Coello & Polleux, 2014; Vingtdeux, Davies, Dickson, & Marambaud, 2011), which may demonstrate a link between energy homeostasis and AD. In a streptozotocin‐induced AD model in rats, AMPK activation by AICAR stopped the cell cycle, improved spatial memory, and ameliorated AD pathology (Du et al., 2015).

Neuroinflammation has been reported in AD. Neuroinflammation markers, such as IFN‐gamma and TNF‐alpha, can also cause cell proliferation. In human AD patients, INF‐gamma, TNF‐alpha, and nitric oxide levels were higher in mild and severe stages than in earlier phases, indicating progressive increases (Belkhelfa et al., 2014). Activated TNF‐alpha and the c‐Jun kinase (JNK) signaling pathway led neuronal cells to progress in the cell cycle toward mitosis, followed by neuronal cell death (Bhaskar et al., 2014). The expression and release of pro‐inflammatory molecules are primarily achieved through glial cells, especially by astrocytes and microglia; glial cell functions are also involved in amyloid‐beta clearance and in neurodegenerative disease conditions, including AD (Pérez‐Nievas & Serrano‐Pozo, 2019; Valles et al., 2019). Consistently, in cerebrospinal fluid, microglia activation marker (soluble triggering receptor expressed on myeloid cells 2 (sTREM)), a marker of microglial inflammatory reaction (monocyte chemoattractant protein‐1 (MCP‐1)), and astroglial activation marker (chitinase‐3‐like protein 1 (YKL‐40)) increased as AD progressed (Nordengen et al., 2019).

### 6.3 High degree of aneuploidy in patients with AD and mild cognitive impairment (MCI)

Human brains are naturally aneuploidogenic during early development and carry a higher rate of aneuploid cells in adulthood. However, compared with healthy subjects, patients with both familial/early‐onset and sporadic late‐onset AD show widespread aneuploidy in their brains and in peripheral blood cells (Bajic, Spremo‐Potparevic, Zivkovic, Isenovic, & Arendt, 2015; Hou, Song, Croteau, Akbari, & Bohr, 2017; Iourov et al., 2009; Lee, Thomas, & Fenech, 2015; Zivković et al., 2010). This AD‐associated aneuploidy is thought to be due to genomic instability (Andriani, Vijg, & Montagna, 2017; Shepherd, Yang, & Halliday, 2018). This observation suggests the existence of a cell population that has newly entered a flawed cell cycle in patients with AD.

### 6.4 Aneuploidy can cause intellectual disability and/or general aging

Many individuals with congenital aneuploidy or aneuploidogenic conditions show symptoms of intellectual disability, suggesting a role of genomic stability in maintaining neuronal and higher cognitive/behavioral functions. Down syndrome (DS) is caused by developmental aneuploidy/chromosome 21 trisomy. DS patients develop early‐onset AD‐like dementia in their 40s‐50s (Lott & Head, 2019; Potter, 1991). The conventional interpretation of the AD‐like dementia in DS patients is that it is due to the APP gene on chromosome 21; triplicate copies of the APP gene facilitate amyloid‐beta accumulation and development of AD in DS patients. Yet, aneuploidy can also produce proteotoxic effects and ER stress, and disturb stoichiometry of macromolecular complexes (Brennan et al., 2019; Chunduri & Storchová, 2019), as well as transcriptomic alterations at the cellular and/or organ level, compared with those of nonaneuploid control subjects (Wangsa et al., 2019). Thus, the general effects of aneuploidy at the cellular and organ levels may contribute to AD development, in addition to APP overproduction in patients with DS. A recent report on a mouse model of triplication of chromosome 21 genes other than APP supports the presence of an effect of aneuploidy on amyloid‐beta accumulation. The mice showed increased amyloid‐beta aggregation and deposition of plaques with cognitive deficits, although they showed no increase in APP abundance (Wiseman et al., 2018).

Cohesinopathy, a defect leading to premature mitotic chromosome segregation and chromosome instability, is an aneuploidogenic condition. Congenital mutations causing cohesinopathy in humans result in conditions with intellectual disability, deformity, and proneness to cancers, such as Cornelia de Lange syndrome and Roberts syndrome (Cucco & Musio, 2016; Zhu & Wang, 2019). Other aneuploidogenic conditions include mosaic variegated aneuploidy syndrome, which is caused by a mutation in (a) a mitotic checkpoint component BubR1/Bub1B (type 1); (b) CEP57, which encodes a centrosomal protein (type 2) (Snape et al., 2011); or (c) TRIP13, which is involved in mitotic checkpoint complex silencing via Mad2 liberation (Alfieri, Chang, & Barford, 2018; Kaisari et al., 2019). A reduction in BubR1 protein in mice (BubR1^H/H^ mice) resulted in a systemic and neuronal progeria condition (Choi et al., 2016) and in impairment of adult hippocampal neurogenesis (Yang et al., 2017). The progeric conditions were partly ameliorated by targeted elimination/reduction of p16INK4‐positive cells (Baker et al., 2011), by enhancing Wnt signaling via loss of Dickkopf‐1 (Seib et al., 2013) or via inhibition of secreted frizzled‐related protein 3 (sFRP3) (Cho et al., 2019), both of which are endogenous Wnt antagonists. These findings suggest that (a) there is a role of genomic maintenance and aneuploidy in aging processes, and that (b) cell proliferation in the hippocampus is controlled by Wnt signaling. This hippocampal defect may affect neurocognitive performance.

### 6.5 AD pathology is associated with mitosis

Amyloid‐beta: Proteases involved in the APP‐to‐amyloid‐beta conversion are increasingly well characterized (Penke et al., 2017; Sikanyika et al., 2019). However, the stage at which APP is converted to amyloid‐beta is a question that is addressed less often. APP can be phosphorylated by multiple kinases, including cdc2, Rho‐associated coiled‐coil kinase 1 (ROCK1), and Polo‐like kinase 2 (Plk2). The phosphorylation affects its proteolytic processing, trafficking, and protein–protein interaction (Muresan & Muresan, 2007; Suzuki & Nakaya, 2008; Vieira, Rebelo, Domingues, da Cruz e Silva, & da Cruz e Silva, 2009). Inhibition of Plk2 reduced Aβ formation, synapse loss, and memory decline in the APP‐swDI AD mouse model (Lee et al., 2019). APP is phosphorylated at Thr668, which occurs during mitosis through mitotic cdc2 kinase (Suzuki et al., 1994). Thr668‐phosphorylated APP was associated with the centrosome (Judge, Hornbeck, Potter, & Padmanabhan, 2011). Lack of APP resulted in delayed G2/mitosis in APP‐/‐ mice (López‐Sánchez, Müller, & Frade, 2005), suggesting a role of APP during mitosis. Moreover, amyloid‐beta generation was increased during mitosis in cycling cultured H4‐15X cells and with antimitotic drug treatments, suggesting that mitotic cells are a source of amyloid‐beta (Judge et al., 2011). Conversely, amyloid‐beta can interfere with mitosis by disrupting mitotic spindles and inhibiting mitotic motors (Borysov, Granic, Padmanabhan, Walczak, & Potter, 2011), suggesting that amyloid‐beta accumulation alone can be aneuploidogenic. Amyloid‐beta oligomer is mitogenic and can trigger the cell cycle (Varvel et al., 2008). Thus, amyloid‐beta accumulation and the aneuploidogenic mitotic state may form an “amyloid‐beta accumulation cycle” (Figure 2a).

Tau: Human neurofibrillary tangles co‐localized with MPM2 antigens, a mitotic marker. The report suggests the involvement of mitosis in generation of tangles (Kondratick & Vandré, 1996). Human neuroblastoma SY5Y cells overexpressing tau provide a model for tauopathy studies. Abnormal tau phosphorylation of the Alzheimer‐type (AT100 immunopositive and Ser422) was observed in these cells during mitosis (Delobel et al., 2002). G2/M blockers (paclitaxel, vinblastine, and vincristine) have a dose‐dependent effect on tau phosphorylation at Ser‐202 and Ser‐396/404 in N2aTau3R cells. The Ser‐201 and Ser‐396/404 phosphorylation on tau are associated with neurofibrillary tangles (Conejero‐Goldberg, Townsend, & Davies, 2008). During mitosis, c‐Jun N‐terminal kinase phosphorylates R406W tau (Tatebayashi et al., 2006). Taken together, these results indicate that mitotic conditions can generate phosphorylated tau, which is associated with neurofibrillary tangles (Pope et al., 1994; Preuss & Mandelkow, 1998; Vincent, Zheng, Dickson, Kress, & Davies, 1998).

### 6.6 Mutations associated with familial/early‐onset AD can cause mitotic error and aneuploidy, as well as other cell cycle disturbances

Presenilin 1/PSEN1 mutation (e.g., familial AD mutation in presenilin 1 [M146L and M146V]) is linked to familial/early‐onset AD. Overexpression of mutant PSEN1 caused chromosome missegregation and aneuploidy in vivo (mice) and in vitro, with mitotic spindle defects observed (Boeras et al., 2008). PSEN1 P117R mutation is a pathogenic AD mutation that can cause increases in p53 and p21 proteins, G1 phase prolongation, S phase shortening, and decreased apoptosis in human lymphocytes (Bialopiotrowicz et al., 2012). Lymphocytes are proposed to serve as a surrogate indicator for the development of AD, as these cells are responsive to oxidative stress and other challenges, and are indicative of aneuploidy and cell cycle disturbances that mirror the condition of neurons in patients with AD (Wojsiat, Prandelli, Laskowska‐Kaszub, Martín‐Requero, & Wojda, 2015). As mentioned in Section 6.5, amyloid‐beta can also disrupt the mitotic spindle and inhibit mitotic motors, thus causing mitotic defects and aneuploidy (Borysov et al., 2011). The APOE subtype is associated with AD risk. Knockdown of APOE in APOE‐expressing ovarian cancer cells led to G2 cell cycle arrest and apoptosis, suggesting its context‐dependent role in cell cycle progression (Chen et al., 2005).

### 6.7 Forced cell cycle re‐entry resulted in amyloid‐beta and p‐tau accumulation in mouse brains

Transgenic mice in which cell cycle reactivation in neurons is forced by SV40 T antigen via the tet‐on/off system show signs of mitotic re‐entry (e.g., PCNA, cyclin B1, MPM2) and Aβ deposits and phosphorylated tau in the brain (Park, Hallows, Chakrabarty, Davies, & Vincent, 2007). Expression of SV40 T antigen causes replication stress, mitotic dysfunction, and aneuploidy (Hu, Filippakis, Huang, Yen, & Gjoerup, 2013), suggesting a link among mitotic re‐entry, aneuploidy, and AD pathology.

### 6.8 Amyloid‐beta can bind to mitotic motors and microtubules, causing mitotic error and aneuploidy, as well as triggering the stress response

Amyloid‐beta can disrupt the mitotic spindle and inhibit mitotic motors (e.g., Eg5, KIF4A, MCAK), causing mitotic defects with prolonged mitosis and aneuploidy (Borysov et al., 2011). The transcriptome of cultured SH‐SY5Y cells expressing P301L tau was most affected in the cell cycle and cell proliferation; proteomic analysis on an amyloid‐beta (1‐42)‐injected mouse model revealed that the stress response and metabolism pathways were most affected (Götz et al., 2008). Amyloid‐beta injection in a rat model caused pro‐apoptotic changes (increased caspase‐3, decreased Bcl2/Bax ratio) and activation of stress/mitogenic signaling (increased pERK, pJNK, and NFkB65kd; decreased IkB) (e.g., Dong, Ji, Han, & Han, 2019). Thus, once accumulated, amyloid‐beta can be aneuploidogenic by itself and can trigger stress response and cell death.

### 6.9 Infection with AD‐associated pathogens can cause mitotic re‐entry, mitotic errors, and/or prolonged mitosis

Various pathogens, including viruses (HHV1‐6, HCV), bacteria (*Chlamydia pneumoniae*, *Helicobacter pylori*), fungi (*Candida albicans*), and protozoa (*Toxoplasma gondii*), have been identified as potential AD risk factors (Sochocka et al., 2017). Herpes simplex virus 1/HSV1/HHV1 immediate–early protein Vmw110 was shown to inhibit G1/S transition and progression through mitosis (i.e., prolonged mitosis at pseudo‐prometaphase), which was in part caused by Vmw110‐induced proteasome‐dependent degradation of a centromeric protein CENP‐C (Everett, Earnshaw, Findlay, & Lomonte, 1999; Lomonte & Everett, 1999). Cytomegalovirus CMV/HHV5 infection caused transcriptomic misregulations in cell cycle and mitosis genes, and produced a pseudo‐mitosis state in the infected cells (Hertel & Mocarski, 2004). *Chlamydia trachomatis* disrupted cytokinesis of the host cells and caused aneuploidy with multinuclei (Sun, Sin, Poirier, & Harrison, 2016). Expression of *Helicobacter pylori* oncoprotein CagA caused (a) uncontrolled cell proliferation by activating the oncoprotein SHP‐2 and (b) spindle misorientation at the onset of anaphase and chromosomal segregation errors with abnormal division axis (Umeda et al., 2009). Phagocytosed *Candida albicans* caused macrophages to fail cell division, leading to large multinuclear aneuploids (Lewis, Bain, Lowes, Gow, & Erwig, 2012). *Toxoplasma gondii* facilitated normally quiescent fibroblasts to enter S phase/mitotic re‐entry, and the effect could be mediated both by direct invasion and by conditioned medium in vitro (Lavine & Arrizabalaga, 2009). These observations of AD‐associated pathogens being able to cause mitotic re‐entry, mitotic errors, and/or prolonged mitosis may help to reconcile the aforementioned “AD is caused by pathogen” theory and the “amyloid‐beta accumulation cycle.”

## 7 WILL ANEUPLOIDY ALONE BE SUFFICIENT TO CAUSE AMYLOID‐BETA ACCUMULATION?

Cohesinopathy–genomic instability model Shugoshin 1 (Sgo1) haploinsufficient mice (Sgo1^−/+^ mice) showed spontaneous cerebral amyloid‐beta accumulation in old age (Figure 2c; Rao, Farooqui, Asch, et al., 2018; Rao, Farooqui, Zhang, et al., 2018). Normally, amyloid‐beta accumulation does not occur in mice. The International Mouse Phenotyping Consortium (IMPC) database reports an abnormal behavior phenotype in Sgo1^tm1a(EUCOMM)Wtsi^ allele mice, suggesting the likelihood of AD‐like cognitive function/behavioral issues with Sgo1 defects (http://www.mousephenotype.org/data/genes/MGI:1919665#section-associations). In the Sgo1^−/+^ mice, we did not observe a higher amount of APP protein. Thus, accumulation of precursor protein APP was unlikely to be the cause of amyloid‐beta accumulation. Amyloidogenic protease BACE and mitotic marker phosphor‐histone H3 co‐localized with amyloid‐beta in amyloid‐beta‐expressing cells, suggesting that mitotic/quasi‐mitotic/mitotic catastrophe cells were responsible for increased amyloid‐beta in aged Sgo1^−/+^ mice (Rao, Farooqui, Zhang et al., 2018).

However, spindle checkpoint defect–genomic instability model BubR1^−/+^ mice did not show cerebral amyloid‐beta accumulation (Rao, Farooqui, Zhang et al., 2018), suggesting that aneuploidy alone may not be sufficient to cause amyloid‐beta accumulation in a mouse model. Since a major difference in these two chromosome instability–aneuploidogenic models is spindle checkpoint function and prolonged mitosis, prolonged mitosis was proposed to be one of the three critical factors (the “three‐hit” hypothesis; Figure 2b) for amyloid‐beta accumulation (Rao, Farooqui, Asch et al., 2018). Thus, types of aneuploidy that are accompanied by prolonged mitosis, such as cohesinopathy and amyloid‐beta poisoning, are speculated to further lead to amyloid‐beta accumulation.

Whether tetraploidization, another type of aneuploidy, contributes to AD development is a matter of controversy. Tetraploidization was reported to occur in normal and AD brains to a similar degree (Westra, Barral, & Chun, 2009). This finding suggests that the effects of tetraploidization on AD development are limited. A newer paper, however, reported a correlation between neuronal tetraploidization in the cerebral cortex in mice and reduced cognition and AD‐associated neuropathology, suggesting a causal role of tetraploidization in the development of AD (López‐Sánchez et al., 2017). For the tetraploidization mechanism, as AD brains abundantly express neurotrophin receptor p75NTR and proNGF (nerve growth factor), their involvement in triggering neuronal tetraploidization, subsequent abortive mitosis, cell death, and hence neurodegeneration was suggested (Frade & López‐Sánchez, 2010). Determining the cause–consequence relationship of tetraploidization in AD may not be simple, as they may occur rather simultaneously.

## 8 THIRTEEN AMONG 37 GENES ON THE HUMAN AD GENETIC RISK LOCI ARE FUNCTIONALLY INVOLVED IN THE CELL CYCLE AND/OR MITOSIS

Analyzing AD brains in a comprehensive and hypothesis‐free manner with a combination of various ‐omics, imaging, and other biomarker analysis techniques has been proposed by the “Alzheimer Precision Medicine Initiative (APMI)” to advance understanding of AD, to identify dysfunctional systems and predictive markers, and to develop remedies against neurodegenerative disorders (Hampel, Toschi, et al., 2018; Hampel, Vergallo, et al., 2018). Genome sequencing projects of human AD patients and meta‐analysis of the reports have revealed genes/loci that are frequently mutated in AD patients, that is, AD genetic risk loci (Beecham et al., 2014; Carrasquillo et al., 2015; Chouraki & Seshadri, 2014; Jansen et al., 2019; Kim, 2018; Kunkle et al., 2019; Lambert et al., 2013; Van Cauwenberghe, Broeckhoven, & Sleegers, 2016; Zhang, Gaiteri, et al., 2013), in addition to known familial AD mutations, such as PSEN1/2, APP, and APOE variants. The genes include 21 previously identified loci: ABCA7, BIN1, CASS4, SORL1, CD33, CD2AP, CELF1, CLU, CR1, DSG2, EPHA1, FERMT2, HLA‐DRB5/HLA‐DRB1, INPP5D, MEF2C, MS4M6A, MS4A4E, NME8, PTK2B/PYK2, SLC24A4, and ZCWPW1. In addition, ADAM10, ACE, NYAP1, SPI1, and ECHDC3 were identified through a recent meta‐analysis (Kunkle et al., 2019). ADAMTS4, HESX1, CLNK, TREM2, CNTAP2, APH1B, KAT8, SCIMP, ABI3, SUZ12P1, ALPK2, and BZRAP‐AS1 were identified with international transethnic cohorts (Jun et al., 2017) (Table 1).

Using genome‐wide association studies (GWASs), Han, Huang, Gao, and Huang (2017) identified functions of the genes and categorized these functions as “regulation of beta‐amyloid formation,” “regulation of neurofibrillary tangle assembly,” “leukocyte‐mediated immunity,” and “protein‐lipid complex assembly” signaling pathways. With the protein–protein interaction network and functional module analyses, they also identified “hub” genes and “bottleneck” genes indicating three subnetworks. The hub genes included APOE, PICALM, BIN1, ABCA7, CD2AP, CLU, CR1, MS4A4E, and MS4A6A, while the bottleneck genes included APOE, TOMM40, NME8, PICALM, CD2AP, ZCWPW1, FAM180B, GAB2, and PTK2B (Han et al., 2017). However, as of 2019, not all genes are well characterized, as indicated by the limited number of publications listed in Table 1.

From supporting evidence of the involvement of the cell cycle and mitotic re‐entry in AD development, we hypothesized that some of the genes identified as AD genetic risk loci are functionally involved in the cell cycle and/or mitosis. We performed a series of literature searches using “cell cycle” or “mitosis” as keywords for each of the genes. The search revealed possible functional involvement in the cell cycle or mitosis regulation for 13 among 37 genes (Table 1). This result provides additional support to the long‐standing hypothesis that human AD development is associated with, influenced by, or caused by misregulations in the cell cycle or mitosis via gene mutations, at least in part. The hypothesis warrants further investigation.

## 9 EFFECTS OF CELL CYCLE INTERFERING DRUGS ON AD MODELS

The aforementioned reports suggest that two major AD pathological features, plaques and tangles, are caused by or associated with cell cycle misregulation toward mitosis. Existing pharmacological reagents can interfere with the cell cycle and its machineries, leading to the question of whether these drugs affect neuronal health and AD symptoms and pathology.

### 9.1 Antimitotic drugs can be neuroprotective against tauopathy

With exceptions of mitotic kinase or mitotic motor inhibitors, most antimitotic drugs target microtubule dynamics and mitotic spindles. Taxanes, including paclitaxel/Taxol, are microtubule stabilizers, while vinblastine and vincristine are microtubule destabilizers. Both classes of antimitotic drugs have a demonstrated history of use in cancer chemotherapy (Florian & Mitchison, 2016). Numerous in vitro results suggest the benefits of antimitotics for tauopathy. For example, cultured Aplysia neurons expressing mutant–human–tau indicate morphological signs of neurodegeneration under live confocal imaging platforms. A clinically relevant dose of 10 nM paclitaxel rescued these effects, whereas 100 nM paclitaxel facilitated them (Shemesh & Spira, 2011). In testing drugs in animal models, blood–brain barrier penetration and cerebral drug availability must also be considered (Brunden et al., 2011). In the PS19 tau transgenic mouse model of tauopathy, the brain‐penetrant antimitotic drug epothilone D reduced the burden of tau pathology (Zhang et al., 2012). Another brain‐penetrant microtubule stabilizer, dictyostatin, also produced improvement in CNS/brain measures (Brunden, Lee, Smith, Trojanowski, & Ballatore, 2017; Makani et al., 2016). Note that most antimitotics target microtubule dynamics. The possibility remains that their neuroprotective effects occur through microtubule and microtubule‐binding protein‐mediated signaling and/or axonal transport, rather than cell cycle effects (Brunden et al., 2017; Trojanowski, Smith, Huryn, & Lee, 2005). There are ongoing translational studies and clinical trials. For example, TPI‐287/abeotaxane is a brain‐penetrant microtubule stabilizer that indicated efficacy in PS19 tau transgenic mice. Recent basket clinical trials on different tauopathies to test the safety, tolerability, and potential efficacy of TPI‐287 infusion indicated that (i) TPI‐287 was generally well tolerated, although anaphylactoid reactions occurred in 3/26 of AD patients, but not in 42 4‐repeat tauopathies (4RT) patients, which led to a discussion of a potential difference in sensitivity profiles of AD and 4RT patients; (ii) CSF YKL‐40 level changed in TPI‐287 groups; and (iii) the AD treatment group showed a smaller decline in MMSE scores than did the placebo group, although the difference was not significant (Tsai et al., 2019). Still, antimitotics remain candidate drugs for AD management.

### 9.2 CDK inhibitors ameliorated AD symptoms in animal AD models

CDK5 is a unique member of the CDK family. Unlike canonical cell cycle driving CDKs, such as CDK1 and CDK2, Cdk5 is inactive in the cell cycle, but is specifically expressed and predominantly active in postmitotic neurons. Its role in AD has long been postulated (Dhavan & Thai, 2001). In physiological conditions, CDK5 binds with its activator, p35, and plays roles in the development of CNS and movements of neurons. However, once neurons experience pathogenic challenges, Cdk5 associates with p25, which is generated from p35 by calpain‐dependent cleavage, and becomes hyperactivated. CDK5/p25 causes aberrant hyperphosphorylation of various substrates that include APP, tau, and neurofilaments. Subsequently, Aβ formation, tau hyperphosphorylation, synaptic plasticity, oxidative stress, and mitochondrial dysfunction occur, followed by neuronal cell apoptosis and neurodegeneration with AD pathology (Liu et al., 2016; Lopes & Agostinho, 2011). CDK5 is also a regulator of other cell cycle regulators, including c‐Jun and p38MAPK (Chang et al., 2010). Misregulation of CDK5 can trigger aberrant activation of cell cycle kinases and phosphatases, leading to neuronal cell death (Chang, Vincent, & Shah, 2012). CDK5 inhibition has a neuroprotective effect (Mushtaq et al., 2016). Diaminothiazoles is a CDK5 and GSK3β inhibitor. Diaminothiazoles decreased PHF‐1 immunoreactivity in two animal models of AD (3xTg‐AD and CK‐p25), showed neuroprotective effects, and helped memory recovery (Zhang, Hernandez, et al., 2013).

Flavopiridol/alvocidib is a potent and specific inhibitor of CDKs 1, 2, 4, and 7 in vitro, showing a clear blockade of cell cycle progression at the G1/S and G2/M boundaries (Senderowicz, 1999). In the hCOX‐2 transgenic mice, overexpression of human COX‐2 in murine primary hippocampal neurons accelerated beta‐amyloid‐mediated apoptosis. The in vitro neuronal damage was prevented by flavopiridol (Xiang et al., 2002). Leggio et al. (2016) tested flavopiridol/alvocidib in an amyloid‐beta‐injected AD mouse model. Aβ‐injected mice showed mitotic cyclin A‐positive cycling neurons in the frontal cortex and the hippocampus, and displayed memory deficits. The cell cycle events and memory deficits were prevented by flavopiridol administered at 0.5 and 1 mg/kg body weight. Zhang, Gaiteri, et al. (2013), reported that (a) a strong association with AD clinical and pathological traits and cell cycle‐enriched module/subnetwork, and (b) the cell cycle‐enriched module/subnetwork showed the second largest loss of gene–gene interactions in AD compared with normal controls, thus was among the most affected. Huang et al. (2019) predicted that denticleless (DTL) is the key driver gene of the cell cycle‐enriched module and investigated the role of DTL‐encoded CDT2 protein in AD pathology. In addition, CDT2 is a part of the cell cycle‐driving CUL4 CRL ubiquitin ligase complex. They reported upregulation of DTL/CDT2 in human patients with AD and increased degradation of p21, a CDK inhibitor protein, in vitro with CDT2 overexpression. In mice overexpressing CDT2, degradation of p21 released CDKs' activity toward cell cycle progression and triggered AD processes, including increases in amyloid‐beta, p‐tau, and BACE; memory deficits; and a gene expression signature similar to that observed in human AD, for example, increases in APOE, CASS4, and CLU. Treatment with CDK2/7/9 inhibitor roscovitine/seliciclib rescued CDT2‐induced cognitive defects in CDT2‐overexpressing mice (Huang et al., 2019).

### 9.3 Targeting mitogenic signaling

Mitogenic signaling is another potential target for AD drugs. However, few clinical trials for AD have explored mitogenic signaling. Those trials included examinations of the following drugs: lactoferrin, which can modulate p‐AKT/PTEN (Mohamed, Salama, & Schaalan, 2019); neflamapimod, a specific inhibitor of p38MAPK‐alpha (Alam, Blackburn, & Patrick, 2017); bryostatin 1, an activator of protein kinase C epsilon (Nelson et al., 2017); tideglusib, an inhibitor of GSK3 (Lovestone et al., 2015); and lithium, a GSK3 inhibitor (Forlenza et al., 2011). Although this “targeting mitogenic kinase/signaling” approach has not become mainstream in AD research and drug development, the approach has been established in cancer research and drug development, with success against oncogene‐addicted cancers (e.g., imatinib/Gleevec targeting Bcr‐Abl tyrosine kinase, trastuzumab/Herceptin targeting Neu receptor). Due to organ‐specific issues (e.g., blood–brain barrier [BBB]‐mediated drug delivery), cancer drug repurposing for AD therapy may not be straightforward. Still, given the abundance of accumulated resources for targeting mitogenic signaling, the approach may hold great future potential.

## 10 CONCERNS TO BE ADDRESSED

Cancer treatments can leave cognitive impairment in a segment of cancer patients during the course of treatment or after completion, a phenomenon known as chemobrain (Argyriou, Assimakopoulos, Iconomou, Giannakopoulou, & Kalofonos, 2011). A meta‐analysis of animal model studies indicated a correlation between cognitive decline and chemotherapy drug treatments, especially with the cisplatin, CMF (cyclophosphamide, methotrexate, fluorouracil combination), and MTX (methotrexate) + 5‐FU chemotherapy regimens (Matsos & Johnston, 2019). However, there is currently little evidence that the treatments caused amyloid‐beta accumulation or triggered AD‐like dementia. Instead, the cognitive decline is attributed to damage to the BBB, oxidative stress, and cytokine dysregulation; thus, this chemobrain phenomenon is believed to be mechanistically closer to vascular dementia (Ren et al., 2019; Ren, St Clair, & Butterfield, 2017). Consistently, severe chemobrain is associated with cytotoxic DNA‐damaging drugs, rather than CDK inhibitors. An inverse relationship between cancer and AD is known; that is, cancer survivors have less likelihood of developing AD (Zhang et al., 2015). The inverse relationship is proposed to be related to a balance in cellular tendencies toward cell death or growth (Shafi, 2016). Mechanisms in cell survival/death regulation, that is, p53, Pin1, and the Wnt signaling pathway, were discussed as potential therapeutic manipulation targets (Behrens, Lendon, & Roe, 2009). In addition to innate cellular tendencies, we speculate that the inverse relationship may be in part due to a therapeutic or intervention effect of cancer chemotherapy drug on preclinical AD. When cell cycle‐managing chemotherapy drugs are to be repurposed for AD prevention and/or therapy, we suggest designing clinical trials with careful and conservative dosing, with a less likelihood of BBB damage and/or chemobrain induction.

## 11 SUMMARY

Increasing evidence, including recent ‐omics data from human patients with AD, points to a critical role of the cell cycle and mitosis, leading to the “amyloid‐beta accumulation cycle,” in the development of AD pathology. CDK inhibitors tested on animal models of AD showed symptomatic relief, corroborating the notion that the cell cycle and mitosis are targets of AD drug research and development. With further support, existing cell cycle and mitosis‐targeting drugs, many of which are clinically used as cancer drugs, may be successfully repurposed as AD drugs in the near future.

## CONFLICTS OF INTEREST

No conflicts of interest declared.

## AUTHOR CONTRIBUTIONS

H.Y. Yamada contributed all aspects of the project. C.V. Rao and A.S. Asch provided intellectual input and material support. D.J.J. Carr contributed generation of unpublished key data.
